# Contemporary management of ‘Inguinal disruption’ in the sportsman’s groin

**DOI:** 10.1186/2052-1847-6-39

**Published:** 2014-11-27

**Authors:** Aali J Sheen, Zafar Iqbal

**Affiliations:** Department of Hernia Surgery, Central Manchester Foundation Trust, Manchester Royal Infirmary, Manchester, M13 9WL UK; Sport’s Medicine, Liverpool FC Training Ground, Melwood Drive, West Derby, Liverpool, L12 8SY U.K

## Abstract

**Background:**

This article helps define the basic principles to diagnosis and manage one of the surgically correctable causes of the ‘painful groin’, which is commonly described as the sportsman’s groin.

**Discussion:**

Often many surgeons will describe a single pathology for the sportsman’s groin such as a ‘hernia’ but often other coexisting etiologies may be present.

Management relies on a multidisciplinary approach with a diagnosis initially made by a history of pain in the groin on exercise. Physiotherapy is the recommended first line treatment and is designed to concentrate on strengthening of the abdominal wall muscle and tendon groups around the groin area.

Surgery does have a role in the sportsman’s groin but only once all conservative measures have been exhausted or if there is a clear identified pathology causing the groin symptoms such as posterior wall defect.

Surgical principles for an inguinal disruption include either open or laparoscopic techniques reinforcing the inguinal canal with a mesh or suture repair followed by an active rehabilitation programme.

**Summary:**

Once an accurate diagnosis has been achieved, contemporary guidance for inguinal disruption requires a multidisciplinary approach including a specially designed physiotherapy regime and possibly surgery.

## Terminology

The commonest presenting symptom that patient’s describe is pain in the groin after exercise [1]. However, the exact nomenclature of this condition has never been very clear with a plethora of descriptions used including the sportsman’s groin (SG), athletic pubalgia, pubic inguinal pain syndrome, an incipient hernia and more recently following a consensus meeting in Manchester the term inguinal disruption (ID) [2–6]. For the purposes of this article the term inguinal disruption will be used.

## Diagnosis

ID is a condition recognized by groin pain and is commonly seen in very active sports persons. It is generally accepted that sports which involve kicking and twisting movements experienced in football (soccer), rugby, fast bowling in cricket and middle distant running are most likely to be affected by this condition with swimmers, cyclists and especially boxers less likely to experience the symptoms of ID [2–5].

A concise literature review reveals that other pathologies may also account for the symptoms of groin pain typically described as ID such as, adductor muscle tendinitis, osteitis pubis, pubic symphysitis, bone marrow oedema and femoro-acetabular impingement (FAI) of the hip joint [7], although it is also accepted that they can coexist with ID [8, 9]. For the general physician and/ or physiotherapist that sees the amateur athlete, ID will be defined as pain in the inguinal region near or below the pubic tubercle. This pain arises either over time or acutely and importantly no obvious previous known pathology exists to explain the symptoms [4, 5].

To help determine the diagnosis clinically, it has been suggested that if at least three out the five signs below exist then a diagnosis of ID can be made [6]:

1. Pin-point tenderness over the pubic tubercle at the point of insertion of the conjoint tendon.
2. Palpable tenderness over the deep inguinal ring.
3. Pain and/or dilatation of the external ring with no obvious hernia evident.
4. Pain at the origin of the adductor longus tendon.
5. Dull, diffuse pain in the groin, often radiating to the perineum and inner thigh or across the mid-line.

The following tests have also been used in addition, with the initial screening of athletes to determine the diagnosis of ID as which, can also be used after any treatment for any improvement or worsening of their symptoms:

1. Weakness on resisted sit ups.
2. Reduced power on adductor squeeze strength.

A thorough clinical examination must always be carried out to assess fully the strength of the adductor & rectus tendon origins, abductor strengths, the gait of the individual as well as to ensure the patient does not have any imbalance as well as confirming there are no entrapment neuropathies [5]. Importantly adductor pain on ‘squeeze test’ may be present in most patients with ID due to the strain that will be elicited around the pubic tubercle. In order to exclude other factors that may contribute to groin pain the clinical assessment and eventual diagnosis must be accompanied with the aid of a suitable imaging modality. Musculoskeletal investigations such as ultrasound or Magnetic resonance imaging (MRI) are important not only to help with the diagnosis but to exclude other pathologies as described above such as adductor tears, hip impingement and chronic osteitis pubis [10–12]. MRI scanning though does not aid in measuring the efficacy of how well surgical or non-surgical treatment has undergone with both symptomatic and asymptomatic athletes demonstrating similar changes on imaging before and after treatment [13]. Bone marrow oedema, osteitis pubis, tendon disruption as well as fluid in the symphysis have all been shown to be present on MRI findings in patients with ID [14].

Ultrasound is a simpler and more easily accessible test and is often used, although it is relatively unhelpful with tendon injuries in the groin as compared to MRI [15]. Ultrasound of the inguinal canal may be normal in athletes with ID but can detect both true inguinal or femoral weaknesses as well as an obvious hernia, but should be undertaken with the patient in both a supine position as well as standing whilst performing a valsalva’s maneuver (stress ultrasound) [15]. Elite athletes may show a posterior wall defect on stress ultrasound but be relatively asymptomatic, but this detected weakness may be used as a possible screening test of the groin ‘preseason’ as well as for new players to achieve a baseline so any subsequent change or worsening can be used to determine if this change is responsible for any ensuing groin pain experienced [15, 16].

In summary a careful and detailed examination combined with an ultrasound and an MRI scan to exclude acute tendon injury or hip pathology is recommended as the minimum for all patients [6].

## Conservative management of inguinal disruption

Once a diagnosis of ID is made, initial treatment is geared towards physiotherapy, analgesia and rest. The large majority of patients will not require any surgical intervention and therefore, be able to return to their chosen sporting activity in the minimum amount of time. The technology and range of physiotherapy has improved vastly over the last twenty years due mainly to an improved understanding of abdominal and gluteal stability of the pelvic girdle with further emphasis placed on effectiveness of the abdominal muscles as well as improving the neglected gluteal and adductors [17].

It is important to seek the expertise of a physiotherapist that has the understanding of the isometric, concentric and eccentric contraction of the musculature around the abdomen, pelvis and hip joint which are then ‘fused’ into functional exercise regimes. Other treatments as well as exercises that can be used include electrotherapy, manual therapy and steroid injections [18].

A randomized trial study compared an active training program with a set physiotherapy regime to no active training for athletes with adductor related groin pain, concluded that the active training program has a significant advantage in returning an athlete to their chosen sport (p = 0.006) [19]. Another study examining the use of a structured exercise program in footballers for the muscles related to the pelvis versus a normal exercise regime also depicted a 31% reduction in the risk of groin injury with the structured program but, this did not reach statistical significance [20]. A recent Cochrane review in 2013 reported the trials examining conservative treatment regimes for groin pain in athletes [18], but could not conclude in recommending any specific treatment regime for athletes with groin pain however, it did report a benefit in the short term in pain relief for ID by use of hip and abdominal muscles strengthening exercises. For patients with ID the multi-modal approach is significantly effective in producing a quicker return to sporting activity with newer treatments such as the use of radiofrequency denervation (RFD) of the inguinal ligament also providing some significant benefit [21].

A multi disciplinary approach involving the physiotherapist, sports physician, radiologist and relevant orthopedic and hernia surgeon is required for non-responsive cases to exclude the various possible etiologies that may contribute to the sportsman’s groin.

In summary core stability exercises as well as programmed physiotherapy regimes are recommended for all patients presenting with ID to try and improve the abdominal and hip muscles and this should be for at least for a minimum of three months and possibly longer in some cases especially if there is good improvement in the pain experienced [6].

## Surgical options

Once conservative measures have failed or been exhausted, groin surgery may have a role in the repair of ID. But importantly, first of all, a detailed history and examination as well as the two imaging modalities described above will aid in determining the type of surgery that is offered [22]. Surgery has historically relied on choosing the operative technique, which the surgeon has the greatest experience in and it is well recognized that hernia surgery is technique driven. One of the controversial aspects of surgery for inguinal disruption is that surgeons perhaps will describe the pathology that ‘suits’ their preferred operation. Hence a notable variety of differing procedures have been described for the same symptoms diagnosing ID [23–28] including a simple suture repair which does not involve the use of a mesh and reporting 67% of professional athletes returning to full activity in a median of 14 days [24]. Keyhole or minimal access repairs reported have included both the totally extraperitoneal (TEP) [25] and the Transabdominal pre-peritoneal (TAPP) [28] approaches. The laparoscopic approaches do report a quicker return to full sporting activity (resumption of main sport) with a 70-90% success rate [25–27]. The question now arises as to what pathology, if any, is seen at the time of surgery? Is the pathology better seen via the laparoscopic technique or open? Also which operation should be used under what circumstances? The open technique or anterior approach can view both the rectus abdominis muscle origin on the pubic bone as well at the conjoint tendon and possibly identify any increased tension on the ilioinguinal nerve and/or genital branch of the genitofemoral nerve, although this finding at the time of surgery may be too subjective [2].

Laparoscopically the ilio-pubic tract, pectineus fascia, origin of the rectus muscle can also be inspected as well as any tears in the iliopectineal ligament with the added advantage of inspection of the contralateral side [25]. One group has suggested that the symptoms of ID relate mainly to the presence of inguinal ligament pathology and describes dividing the inguinal ligament via the keyhole technique followed by mesh reinforcement [27].

A much-needed randomized controlled trial comparing both open and laparoscopic techniques may provide an answer to the best-suited operation for ID. Surgery though does appear to have an advantage with one study demonstrating that a TEP repair was better than non-operative management for athletic pubalgia with an earlier return to sporting activity in the operative group (90% to 27% (p < 0.001) [9].

Advocates of minimal access and open surgery will continually debate upon which operation is superior. Many surgical techniques have been described with good effective results as discussed earlier but no two operations have been compared to date for ID. Should the contralateral asymptomatic groin have surgery? This question remains unanswered with only 5% of surgeons in a recent survey of a large group of European Surgeons, operating on the contralateral side routinely [29]. Only 3% of athletes complain of recurrent groin pain within five years as reported from the largest series with 10% complaining of pain on the non-operative side within 10 years [23].

Once surgery has been undertaken it is important to ensure that the patient undergoes a suitable rehabilitation programme, which should be designed to suit the demands of the sport/occupation [6]. The majority of athletes should be able to returned to their chosen sport after a period of training and rehabilitation in 4–8 weeks from the date of surgery.

A common question arises as to whether similar or different injuries are seen comparing amateur with elite athletes and whether they require any different assessment or treatment? In essence both elite and amateur athletes experience the same symptoms with similar signs detected on examination. To date there has been no study comparing directly groin pain in elite and amateur athletes. Elite athletes tend to present at a much younger age by virtue of the fact that they have been training and competing from an earlier age. One study demonstrated a small but not significant, earlier return to their chosen sporting activity [27]. This latter fact could be explained by the fact that elite athletes are physiologically ‘fitter’ than amateur athletes, as well as having better access to physiotherapy. Amateur athletes also tend to have more chronic pathology and again this may be related to poorer access to both physiotherapy and surgery.

In summary surgery is based on experience and expertise and should ideally be undertaken by surgeons, whether open or laparoscopic, by a suitably trained surgeon in the field of ID [23–26].

## Summary

Inguinal disruption is a diagnosis, which is made with a careful history and by eliciting at least 3 out of 5 suggested clinical indicators on examination. An ultrasound can aid in the diagnosis with MRI imaging of the groin used to mainly exclude other differentials from the hip and adductor tendons. However, once an accurate diagnosis has been made conservative measures should always be the first line treatment with surgery eventually considered to reinforce the inguinal canal by either the open minimal repair or TEP techniques, which, are the commonest methods used in studies to date. Management for both elite and amateur athletes is essentially the same with possibly elite athletes able to recuperate more quickly.
